# Characterizing Active Site Conformational Heterogeneity along the Trajectory of an Enzymatic Phosphoryl Transfer Reaction

**DOI:** 10.1002/anie.201606238

**Published:** 2016-08-18

**Authors:** Cathleen Zeymer, Nicolas D. Werbeck, Sabine Zimmermann, Jochen Reinstein, D. Flemming Hansen

**Affiliations:** ^1^ Department of Biomolecular MechanismsMax Planck Institute for Medical ResearchJahnstrasse 2969120HeidelbergGermany; ^2^ Division of BiosciencesInstitute of Structural and Molecular BiologyUniversity College LondonGower StreetLondonWC1E 6BTUK

**Keywords:** active-site dynamics, arginine, conformational entropy, NMR spectroscopy, nucleoside monophosphate kinase

## Abstract

States along the phosphoryl transfer reaction catalyzed by the nucleoside monophosphate kinase UmpK were captured and changes in the conformational heterogeneity of conserved active site arginine side‐chains were quantified by NMR spin‐relaxation methods. In addition to apo and ligand‐bound UmpK, a transition state analog (TSA) complex was utilized to evaluate the extent to which active site conformational entropy contributes to the transition state free energy. The catalytically essential arginine side‐chain guanidino groups were found to be remarkably rigid in the TSA complex, indicating that the enzyme has evolved to restrict the conformational freedom along its reaction path over the energy landscape, which in turn allows the phosphoryl transfer to occur selectively by avoiding side reactions.

Enzymes catalyze chemical reactions by reducing the free energy difference between ground and transition state(s)^1^ and in‐depth characterizations of enzymatic transition states therefore become essential for understanding mechanisms of enzymes. Approaches utilizing kinetic isotope effects, linear free energy relationships, X‐ray crystallography, and computational modeling have divulged key features of transition states.^2^ However, experimental information on the entropic contribution to the transition state free energy originating from conformational heterogeneity is still strikingly lacking.^3^ Whereas the transition state of a simple chemical reaction may be a single well‐defined saddle point on the potential energy surface, enzyme‐catalyzed reactions have been suggested to exhibit an ensemble of transitions states, thus reflecting the dynamic nature of enzymes.^4^ In such scenarios, characterizing the variation in heterogeneity along the reaction coordinate becomes critically important for understanding enzymatic function since heterogeneity relates to entropic (de)stabilization of the transition state(s).

Nucleoside monophosphate (NMP) kinases, which catalyze reversible phosphoryl transfer reactions between ATP and a nucleoside monophosphate, provide an excellent system for investigating the enzymatic stabilization of transition states.^5^ Crystal structures of NMP kinases in several states have shown substantial conformational changes upon substrate binding and revealed the enthalpic stabilization along the reaction coordinate,^6^ including the transition state of the chemical phosphoryl transfer step (Figure 1).^7^ Also, backbone dynamics of the NMP kinase adenylate kinase were characterized using NMR spectroscopy as well as computational approaches^8^ with emphasis mainly on the large‐scale conformational exchange associated with the opening of the nucleotide binding lid, which was elegantly identified as the rate‐limiting step for the overall reaction.^9^

**Figure 1:**
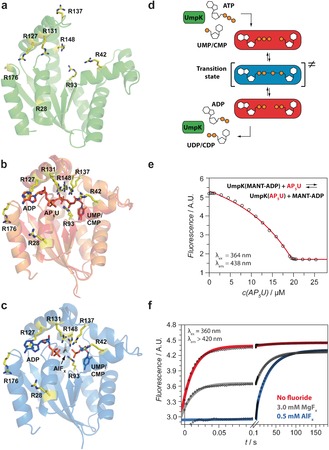
a) Nucleotide‐free UmpK (*H. sapiens*, PDB: 1TEV^10^). b) Overlay of UmpK in complex with the bi‐substrate inhibitor AP_5_U, (red, *D. discoideum*, PDB: 1UKE^11^), and in complex with ADP and CMP (orange, *D. discoideum*, PDB: 2UKD^7^). c) UmpK in complex with the TSA ADP:AlF_x_:CMP (*D. discoideum*, PDB: 3UKD^7^). In (a–c) arginine side‐chains are shown in yellow (numbering refers to UmpK from *D. discoideum*). d) Schematic representation of the reaction catalyzed by UmpK and the states studied here: nucleotide‐free (green), substrate/product‐bound (red), and the transition state (blue). e) AP_5_U binding to UmpK monitored by equilibrium displacement of fluorescent MANT‐ADP. Binding of AP_5_U (*K*_D_≤1.0 nm) generates a stable substrate/product‐like state. f) Nucleotide exchange kinetics measured by stopped flow. UmpK incubated with UMP and ADP in the presence of 0.5 mm AlF_*x*_ (blue), 3 mm MgF_*x*_ (grey) or in the absence of any fluoride (red) was mixed rapidly with the competing ligand MANT‐AP_5_A‐MANT. Notably, the dissociation of ADP and UMP is 2000‐fold slower in the presence of AlF_*x*_ (*k*_slow_≈0.03 s^−1^) than in the absence of metal fluoride (*k*_fast_≈60 s^−1^) and the complex with AlF_*x*_ is characterized exclusively by slow nucleotide exchange.

Here we focus on the central phosphoryl transfer reaction step of NMP kinases, using the UMP/CMP kinase (UmpK) from *Dictyostelium discoideum*, and turn the attention directly to the conformational heterogeneity of the catalytic site and the functional groups that actively participate in stabilizing the transition state, namely the positively charged side‐chains of the six conserved arginine residues^5^ (Figure 1). Since the guanidino groups of these arginine residues, along with the Walker lysine and the catalytic Mg^2+^, form the catalytic site of UmpK and the highly homologous adenylate kinase, the heterogeneity of the catalytic site is well captured in the heterogeneity of the conserved arginine side‐chains.

First, we defined and characterized stable states along the reaction coordinate (Supporting Information). One reference state is nucleotide‐free (*apo*) UmpK (Figure 1 a), where the catalytic side‐chains are expected to be very flexible because of the absence of any ligand, thus giving an indication of the upper limit of conformational entropy. An ideal reference for a substrate/product‐bound state is provided by UmpK in complex with the bi‐substrate inhibitor AP_5_U (Supporting Information; Figure 1 b), where a phosphate group covalently links ATP and UMP such that nucleotide‐like binding occurs but without turnover.^11^ The overall structure of UmpK in complex with AP_5_U is essentially identical to other nucleotide‐bound structures available (Figure 1 b). Despite minor differences in the lid loop conformation and the aliphatic part of the R137 side‐chain, the position of the central guanidino groups is nearly identical. Moreover, fluorescence titrations show that a very stable complex between AP_5_U and UmpK is formed with nanomolar affinity (Figure 1 e), thereby altogether substantiating that the AP_5_U complex is a good reference for a substrate/product‐bound state. The transition state of the phosphoryl transfer reaction is well‐represented by UmpK in complex with the TSA formed by ADP, UMP/CMP and an AlF_*x*_ molecule that mimics the phosphoryl group being transferred (Figure 1 c).^7^ To further confirm the formation of a stable complex that captures the transition state structure, we performed functional studies including nucleotide exchange kinetics measurements in the presence and absence of aluminium fluoride (Figures 1 f and S1/S2, Table S1).

The six conserved arginine residues in the active site of NMP kinases (R42, R93, R127, R131, R137 and R148) are essential for substrate binding and catalysis,^5^ and these form the basis for our NMR‐based analysis of the heterogeneity of the active site. For the three different states of UmpK described above arginine ^1^H_*ϵ*_‐^15^N_*ϵ*_ and ^13^C_ζ_‐^15^N_*ϵ*_ NMR spectra were obtained (Figure 2b). These two sets of spectra are complementary since resonances that are weak or invisible in the proton‐detected ^1^H_*ϵ*_‐^15^N_*ϵ*_ spectra, mainly because of solvent‐accessibility of the arginine side‐chain, often give rise to strong peaks in the carbon‐detected ^13^C_ζ_‐^15^N_*ϵ*_ spectra.^12^ Arginine‐to‐lysine mutations were used to assign the eight arginine side‐chains of nucleotide‐free UmpK and AP_5_U‐bound UmpK, simply by observing peaks disappearing in the NMR correlation spectra (Figure S3). However, this approach fell short for the TSA complex as most mutations impaired the formation of this sophisticated state. The arginine side‐chain chemical shifts were therefore assigned via backbone resonances of the TSA complex, which in turn were obtained using standard triple‐resonance NMR experiments (see Supporting Information).

**Figure 2:**
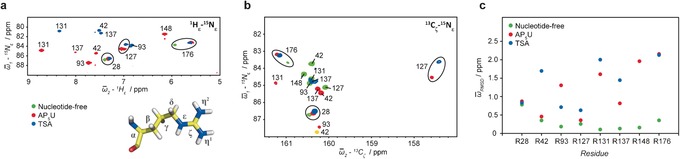
a) ^1^H_*ϵ*_‐^15^N_*ϵ*_ and b) ^13^C_ζ_‐^15^N_*ϵ*_ NMR spectra and chemical shift assignments of the arginine side‐chains: Nucleotide‐free UmpK (green), UmpK in complex with AP_5_U (red), and UmpK in complex with the TSA (blue). A detailed description of the assignment strategy is given in Supporting Information (see also Figure S3). The resonance assigned to R42 in the TSA ^13^C_ζ_‐^15^N_*ϵ*_ spectrum is folded and therefore appears in yellow. c) Deviation of the assigned ^15^N_*ϵ*_ and ^13^C_ζ_ chemical shifts from random coil values. Shown is the $\bar{\omega }$_RMSD_ $=\sqrt{1/2\sum {\left(\delta_{i}-\delta_{RC,i}\right)}^{2}/\sigma_{\delta }^{2}}$ , where *δ*_i_ is the assigned chemical shift, *δ*_RC,*i*_ is the random coil chemical shift, and *σ* is its standard deviation (from the BMRB database).

The obtained correlation spectra show substantial chemical shift differences for the catalytic arginine side‐chains in the three states and a comparison of the assigned chemical shifts to those expected for a “random coil state” shows that the arginine side‐chains in the nucleotide‐free state are all very flexible, whereas a more defined structure is observed in both the AP_5_U and TSA complex (Figure 2 c).

NMR spin‐relaxation methods were used to characterize the heterogeneity of the arginine side‐chains at a level whereby conformational entropy can be estimated. An initial reporter of pico‐nanosecond heterogeneity is the transverse relaxation rate measured in the rotating frame, *R*_1ρ_(^15^N_*ϵ*_). The lower values obtained for the nucleotide‐free state compared to the AP_5_U and TSA complexes (Figure 3 a) indicate a high level of conformational heterogeneity for the catalytic arginines in the nucleotide‐free form and a high level of rigidity in the AP_5_U and TSA complexes, which is in agreement with the chemical shift analysis.

**Figure 3:**
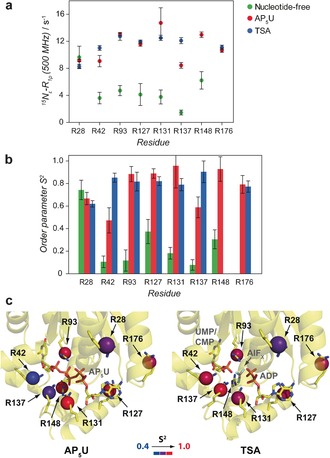
a) Relaxation rates in the rotating frame, *R*_1ρ_(^15^N_*ϵ*_), for UmpK arginine side‐chains in the nucleotide‐free form (green), AP_5_U complex (red) and TSA complex (blue). For the TSA complex the relaxation rates were derived from the ^1^H‐detected experiments, for the AP_5_U complex the rates were derived from both the ^13^C‐detected and the ^1^H‐detected experiments, whereas for the nucleotide‐free form the rates were derived from ^13^C‐detected experiments only (Supporting Information). b) Order parameters *S* ^2,^ describing pico‐nanosecond motions of UmpK arginine side‐chains in the nucleotide‐free form (green), AP_5_U complex (red) and TSA complex (blue), were obtained from global analyses of the available ^15^N_*ϵ*_ relaxation data (Table S2) using the model‐free approach.^13^ c) Conformational heterogeneity of UmpK arginine side‐chains in the AP_5_U complex (left) and in the TSA complex (right). The order parameters *S* ^2^ shown in (b) were mapped on the respective crystal structures (AP_5_U, PDB: 1UKE;^11^ TSA, PDB: 3UKD^7^). All parameters obtained from the global analysis of the relaxation data are given in Table S3.

A more quantitative reporter on the conformational heterogeneity of the arginine side‐chains is the squared order parameter, 0<*S* ^2^<1, of the ^15^N_*ϵ*_−^1^H_*ϵ*_ bond. The order parameter reports directly on the amplitude of local pico‐nanosecond motions and can be obtained from NMR ^15^N_*ϵ*_ relaxation data, whereas changes in the femtosecond and low picosecond dynamics of the side‐chains are not assessed. An order parameter close to unity, *S* ^2^≈1, shows that the arginine side‐chain in question is rigid with respect to the overall enzyme and *S* ^2^≈0 shows that the side‐chain is completely flexible. Consequently, the order parameters *S* ^2^, obtained from analyzing ^15^N_*ϵ*_ relaxation data of the active‐site arginine residues, are directly related to the conformational heterogeneity of these catalytically essential chemical groups, thereby allowing an estimation of the active site conformational entropy.

For UmpK, site‐specific *S* ^2^ (Figure 3c) were obtained from a combined analysis of *R*_1_(^15^N_*ϵ*_), *R*_2_(^15^N_*ϵ*_) and *R*_dd_(^15^N_*ϵ*_‐^1^H_*ϵ*_) relaxation rates obtained at several magnetic field strengths and using the model‐free analysis.^13^ The transverse relaxation rates, which form the basis for obtaining the order parameters, can have contributions from both pico‐nanosecond and slower micro‐millisecond exchange dynamics. A set of exchange‐free relaxation rates, *R*_dd_, that yield the pure dipole–dipole ^15^N transverse relaxation rate was therefore included^14^ to separate the pico‐nanosecond motions from the micro‐millisecond exchange dynamics, thereby allowing accurate order parameters to be obtained. Thus, changes in the order parameters obtained here mainly report on changes in the sampling of side‐chain dihedral angles. The small contributions from micro‐millisecond exchange, *R*_ex_<2.5 s^−1^ at 11.7 T (Table S3), observed for the AP_5_U and the TSA complexes, have previously been associated with lid domain motions upon substrate binding and product release and indicate the presence of minor populated conformations. However, the small values of *R*_ex_ observed here have so far precluded a quantitative interpretation and we therefore concentrate on the order parameters *S* ^2^ for an analysis of the catalytic site heterogeneity in what follows.

In the TSA complex all the catalytic side‐chains are fully locked (⟨*S* ^2^⟩=0.84±0.06) into positions that are optimal for stabilizing the negative charges emerging during phosphoryl transfer, which shows that rigid functional groups and a narrow transition state ensemble are preferential. Significantly lower order parameters (*S* ^2^<0.6) are observed in the AP_5_U state for the side‐chains of R42 and R137 (Figure 3 b), which are both in the vicinity of the UMP/CMP‐site. This observation agrees with the chemical shifts and their deviation from random coil (Figure 2 c). Overall, whereas some of the catalytic arginine side‐chains show conformational heterogeneity in the complex with the tight‐binding substrate‐like ligand AP_5_U, all active site arginine side‐chains are rigid in the TSA complex (Figure 3 c).

Changes in order parameters of several amino acid side‐chains and the protein backbone have previously been used to estimate changes in conformational entropy of proteins.^15^ The active site of UmpK is predominantly formed by arginines and we therefore adapted an approach, where changes in the order parameter of the catalytic arginine side‐chains are used to probe changes in conformational entropy of the active site. The origin for such an interpretation is that the flexibility of the side‐chain, which gives raise to lower order parameters, contributes to the conformational entropy. Two types of side‐chain motions are in general considered 1) intra‐well motion and 2) inter‐well motions that correspond to jumps between different rotameric states. Whereas the intra‐well motion is not expected to vary significantly between different states, the inter‐well motions, the so‐called *J*‐band motions, are good reporters of the conformational entropy.15a

The ^15^N_*ϵ*_‐^1^H_*ϵ*_ order parameter is not sensitive to all arginine side‐chain motions, since there exist multiple conformations of the side‐chain that have a similar orientation of the H_*ϵ*_−N_*ϵ*_ bond vector.^16^ However, although a strict relationship between the order parameter and conformational entropy is not available, important information about conformational entropy can be obtained from the order parameters. For an arginine side‐chain changing from random coil populations to a fully rigid state^17^ one can calculate according to the second term of Eq S9 that the change in conformational entropy is −*TΔS*_conf_≈8.1 kJ mol^−1^. To obtain a more quantitative picture of the relation between side‐chain rotameric populations, conformational entropy, and order parameters we simulated the rotameric sampling of the four dihedral angles of an arginine side‐chain and calculated conformational entropy (term 2 of Eq. S9) as well as the order parameter (Eq. S10). The result of the simulation is shown in Figure 4.

**Figure 4:**
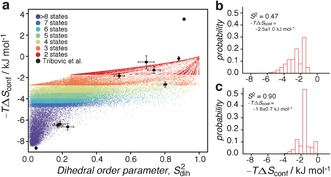
a) Simulated conformational entropy versus side‐chain order parameter, obtained at a temperature of *T=*298 K and for the simulation described in the Supporting Information. The data are colored according to how many states are sampled of the 34 possible arginine side‐chain rotamer states.^[17]^ Shown are 80 000 randomly selected data points out of the totally 8×10^6^ obtained. Included as black points are the correlations obtained previously from MD simulations of RNaseH^16^ (see Supporting Information). b) Histogram showing the distribution of conformational entropies for an order parameter of 0.46<*S* ^2^<0.48. c) Histogram showing the distribution of conformational entropies for an order parameter of 0.89<*S* ^2^<0.91. In (b) and (c) all 8×10^6^ points were used.

The simulations allow us to estimate the change in conformational entropy between the TSA complex, the AP_5_U complex and the nucleotide‐free state. If the motions of the six arginine side‐chains in the states are uncorrelated the change in conformational entropy can be calculated as the sum of each of the individual six side‐chains. For example, the order parameter of R42 changes from 0.11±0.05 to 0.47±0.11 between the nucleotide‐free state and the AP_5_U complex, which can be translated into a change in conformational entropy of 3.2±1.8 kJ mol^−1^. Summing over the six catalytic‐site arginine side‐chains gives −*T*(*S*_AP5U_−*S*_Free_)=18±4 kJ mol^−1^.

In contrast to the change between nucleotide‐free and ligand‐bound, there is only a very small entropic destabilization of the transition state compared to the substrate‐bound state. The order parameter of R42 changes from 0.47±0.11 (Figure 4 b) to 0.85±0.04 between the AP_5_U state and the TSA complex, whereas the order parameter of R137 changes from 0.59±0.09 to 0.90±0.10 (Figure 4 c). Thus, using the distributions of Figure 4, a change in conformational entropy can be calculated to be −*TΔS*=0.9±0.4 kJ mol^−1^ for R42 and 0.7±0.3 kJ mol^−1^ for R137. Assuming the motions of the side‐chains are uncorrelated, the total change in conformational entropy is −*T*(*S*_TSA_−*S*_AP5U_)=1.1±2.8 kJ mol^−1^ (using R42, R93, R127, R131, R137). It is worth noticing that the change in conformational entropy would be even smaller in the event that the motions of side‐chains are concerted. Only considering the contribution from the catalytic arginine side‐chains, a significant entropic cost is paid upon substrate binding, since these side‐chains are close to random‐coil flexibility in the nucleotide‐free state, while nearly rigid in the ligand‐bound states. A very small entropic change is calculated between the TSA complex and the AP_5_U complex (Figure 5).

**Figure 5:**
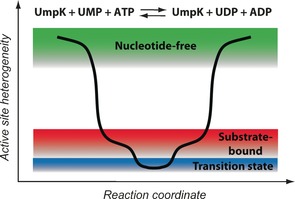
Schematic representation of the heterogeneity of the UmpK active site along the phosphoryl transfer reaction.

The reaction rate of the chemical phosphoryl transfer step is determined by the total difference in free energy between ground and transition state, which includes not only changes within the catalytic site, but also in rest of the protein as well as the solvent. It is therefore of interest to compare the entropic change obtained here for the catalytic site with the expected overall change in entropy. The entropic barrier for a few kinases, where the phosphoryl transfer step is rate‐limiting, has been characterized previously (homoserine kinase: 0.84 kJ mol^−1^, hexokinase: −2.1 kJ mol^−1^, *N*‐acetylgalactosamine kinase: −13.4 kJ mol^−1^).^18^ Thus, the small entropic contribution to the transition state free energy found previously is in good agreement with the small changes in active site conformational entropy observed here.

A general challenge faced by all kinases is the unproductive hydrolysis of ATP. The enzymes have therefore evolved to efficiently suppress this side reaction for example by tightly coordinating the water molecules in the active site.^19^ Tight coordination of water may be achieved by reducing the conformational heterogeneity of functional groups as observed here for UmpK (Figure 3 and 5). The potentially small entropic penalty paid by rigidifying the transition state of UmpK, compared to the substrate‐bound state, could therefore be well worth if it allows the phosphoryl transfer to occur selectively and eliminates side reactions.

The possible importance of an ensemble of transition states and multiple paths over the energy barrier has been discussed for decades,^20^ however experimental methods to directly and site‐specifically probe the corresponding features of the transition state(s) have so far been limited. Our work offers an experimental strategy to assess the conformational sampling of catalytically essential groups in states along the reaction coordinate.

## Experimental Section

Samples of UmpK were expressed and purified as described in the Supporting Information. Nucleotide exchange kinetics experiments were performed by using a stopped flow instrument (see Supporting Information). NMR relaxation experiments were performed on Bruker Avance III 500 MHz (RT‐TXI probe), 600 MHz (CP‐TXO probe), 700 MHz (CP‐TCI probe) spectrometers. NMR spectra were processed and analyzed as described previously^12^ and as detailed in Supporting Information. A comprehensive list of all experiments including sample details and experimental conditions is given in the Supporting Information.

## Supporting information

As a service to our authors and readers, this journal provides supporting information supplied by the authors. Such materials are peer reviewed and may be re‐organized for online delivery, but are not copy‐edited or typeset. Technical support issues arising from supporting information (other than missing files) should be addressed to the authors.

Supplementary
