# Supplementary material for: Characterizing Active Site Conformational Heterogeneity along the Trajectory of an Enzymatic Phosphoryl Transfer Reaction
Source: Angew Chem Int Ed Engl. 2016 Aug 18;55(38):11533–7. doi: 10.1002/anie.201606238 (PMC5026167; doi:10.1002/anie.201606238)
Supplement: Supplementary file 1 — Supplementary [file ANIE-55-11533-s001.pdf]

Angewandte Chemie
Eine Zeitschrift der Gesellschaft Deutscher Chemiker

Supporting Information

# Characterizing Active Site Conformational Heterogeneity along the Trajectory of an Enzymatic Phosphoryl Transfer Reaction

*Cathleen Zeymer[+], Nicolas D. Werbeck[+], Sabine Zimmermann, Jochen Reinstein,* and D. Flemming Hansen**

anie_201606238_sm_miscellaneous_information.pdf

Table of Contents

**Materials and Methods…………………………………………………………………… 3**

1. Mutagenesis, protein expression and purification
2. Fluorescence titrations and nucleotide exchange kinetics measurements
3. Defining stable reference states along the phosphoryl transfer reaction catalyzed by UmpK
4. Sample preparation for NMR experiments
5. Chemical shift assignment of the arginine side-chains of UmpK
6. Nuclear spin-relaxation measurements
7. Estimation of conformational entropy from arginine side-chain order parameters

**Supplementary Figures………………………………………………………….......  13**

Figure S1. Cooperative nucleotide binding

Figure S2. Aluminium fluoride ($AlF_x$) titration

Figure S3. Representative NMR correlation spectra of arginine-to-lysine mutants of UmpK

Figure S4. Consistency of derived order parameters

**Supplementary Tables…………………………………………………………......... 17**

Table S1. Cooperative nucleotide binding

Table S2. $^{15}N_\varepsilon$ relaxation data of UmpK

Table S3. Order parameters $S^2$, local correlation times $\tau_e$ and exchange contributions $R_{ex}$

**References………………………………………………………………………………… 20**

**Materials and Methods:**

**1. Mutagenesis, protein expression and purification:**

Arginine to lysine mutations were introduced into UmpK from *Dictyostelium discoideum* using the QuikChange® protocol. DNA sequencing was performed to verify the eight introduced mutations. Unlabeled [U-$^{1}$H,$^{12}$C,$^{14}$N] UmpK was expressed in LB medium, uniformly $^{15}$N-/$^{13}$C-labeled [U-$^{13}$C,$^{15}$N] UmpK was expressed in M9 minimal medium using 2 g/L U-$^{13}$C-glucose and 1 g/L $^{15}NH_4Cl$ as the sole carbon and nitrogen sources, respectively. Selectively labelled [$^{13}$C,$^{15}$N-Arg; U-$^{12}$C,$^{14}$N] UmpK and [$^{13}$C,$^{15}$N-Arg+Lys; U-$^{12}$C,$^{14}$N] UmpK with only (*i*) arginine residues or (*ii*) lysine and arginine residues isotopically labelled, respectively, were expressed in M9 minimal medium supplemented (*i*) 100 mg/L $^{15}$N-/$^{13}$C-arginine or (*ii*) 100 mg/L $^{15}$N-/$^{13}$C-arginine and $^{15}$N-/$^{13}$C-lysine. All UmpK variants were purified as described previously using Blue Sepharose affinity chromatography and SourceQ anion exchange chromatography[1].

**2. Fluorescence titrations and nucleotide exchange kinetics measurements:**

***Fluorescence titrations:*** Ligand binding to UmpK wild-type and R93K were quantified using fluorescence equilibrium titrations. While binding of U- and C-nucleotides gave rise to a change in intrinsic tryptophan fluorescence and could be observed directly ($\lambda_{ex}$= 296 nm, $\lambda_{em}$= 330 nm), fluorescently labelled MANT-ADP was utilized to obtain binding affinities of ADP, ATP and $AP_5U$ by displacement titrations ($\lambda_{ex}$= 364 nm, $\lambda_{em}$= 438 nm). All measurements were carried out at 25 °C in buffer A (50 mM Tris/HCl pH 7.5, 20 mM KCl and 1 mM TCEP) using a JASCO FP-8500 fluorescence spectrometer. Titrations were performed in the presence of either 5 mM $MgCl_2$ or 10 mM EDTA ("Mg-free").

In order to investigate the cooperativity of nucleotide binding to UmpK, U- and C-nucleotides were titrated in the absence and presence of A-nucleotides and *vice versa*. UMP, CMP, UDP or CDP were titrated into 5 µM UmpK in the presence and absence of 250 µM ADP. $K_D$(MANT-ADP) was determined by titrating UmpK into 3 µM MANT-ADP in the presence and absence of 250 µM CMP. The quadratic equation for ligand binding was used for data fitting[2]. Displacement titrations were performed to determine the binding affinities of ADP, ATP and $AP_5U$. Here, 25 µM UmpK was incubated with 5 µM MANT-ADP and subsequently titrated with ADP, ATP or $AP_5U$, respectively. The cubic equation for binding of competing ligands was used for data fitting[3]. Displacement titrations were performed in the presence and absence of 250 µM CMP.

***Nucleotide exchange kinetics:*** A functional characterization of the transition state analog (TSA) complex (UmpK:ADP:$AlF_x$:UMP) was performed by measuring nucleotide exchange kinetics on a BioLogic SFM 400/MOS 450 stopped flow instrument in single mixing configuration equipped with a 1.5 x 1.5 mm quartz cuvette (FC 15) at 25 °C in buffer A. The dissociation of the TSA complex was monitored by binding of the fluorescent ligand MANT-$AP_5A$-MANT. The excitation wavelength was set to 360 nm, emission was monitored using a 420 nm long pass filter (420FG03-25, LOT Oriel Group). Typically, three to five traces were recorded and averaged. 4 µM UmpK was incubated with 500 µM UMP and 250 µM ADP in the presence of either 0.5 mM $AlF_x$, 3 mM $MgF_x$ or in the absence of any metal-fluoride and then mixed rapidly with 10 µM MANT-$AP_5A$-MANT. The $AlF_x$ stock solution was prepared from $AlCl_3$ and NaF in 1:5 molar ratio at pH 6. All buffers contained 3 mM $MgCl_2$, thus, $MgF_x$ conditions were realized by simply adding 10 mM NaF. The resulting time traces were fitted with the sum of two exponentials to account for MANT-$AP_5A$-MANT binding to the UmpK:ADP:UMP complex (fast phase) and to the UmpK:ADP:$AlF_x$:UMP complex (slow phase).

Nucleotide exchange kinetics were measured at different concentrations of aluminium fluoride (0, 20, 50, 100, 300, 500 and 1000 µM). A plot of the resulting amplitudes for the fast phase *versus* the aluminum concentration and analysis with a quadratic equation isotherm indicates the apparent affinity of aluminium fluoride in the given TSA complex.

**3. Defining stable reference states along the phosphoryl transfer reaction catalyzed by UmpK:**

We sought to define three representative states along the phosphoryl transfer reaction of UmpK, that is, (1) a nucleotide-free state, (2) a substrate/product-bound state and (3) the transition state. Nucleotide-free UmpK was stable during our experiments and this form provided the first reference state.

***Substrate/product-bound state:*** In principle, an active complex like UmpK:ATP:UMP or UmpK:ADP:UDP should allow for assessment of mobility of catalytic residues in a substrate/product-bound state. We had to reach the conclusion however, that such complexes are not amenable to our analysis of active site heterogeneity. Firstly, the high concentrations of UmpK and the rather long measurement times (days) needed for NMR experiments caused minor side reactions to be detrimental for a stable composition of nucleotides (HPLC analysis, data not shown). Secondly, it would be virtually impossible to deconvolute the intrinsic dynamics of the catalytic site with dynamics of an ongoing reaction that includes

opening/closing of the nucleotide-binding lid and other structural adjustments to the nucleotide occupancy.

We also tested the possibility of creating a representation of a substrate-bound state by a UmpK$^{\dagger}$:UMP:ATP and/or UmpK$^{\dagger}$:UDP:ADP complex, where UmpK$^{\dagger}$ symbolizes an inactive form of UmpK. We considered such a strategy by removing the catalytically important $Mg^{2+}$ ion with EDTA, which renders UmpK (nearly) inactive. However, slow but significant turnover was observed, which would interfere with our NMR experiments. Furthermore, the loss of cooperativity in nucleotide binding in the absence of $Mg^{2+}$ indicates that the functional conformation of the active site is at least partially impaired (**Table S1**).

We therefore arrived at the conclusion that the best reference for a substrate/product-bound state is provided by a complex between UmpK and the bi-substrate inhibitor $AP_5U$, in which a phosphate links ATP and UMP (or ADP and UDP) covalently, such that nucleotide-like binding occurs but without turnover.

***Transition state:*** There is an on-going debate in the literature on whether the molecule that mimics the transferring phosphoryl group in various kinase:TSA complexes is an aluminium or a magnesium fluoride species. The planar tetragonal or trigonal molecules observed in TSA crystal structures at different pH were initially interpreted as $AlF_4^-$ or $AlF_3$ species[4], respectively, but there is evidence that at neutral and high pH, trigonal $MgF_3^-$ may be the dominant species, as it provides a favorable negative charge[5]. We addressed this matter by measuring nucleotide exchange kinetics in the presence of magnesium fluoride under aluminium-free conditions and found a biphasic behavior (**Figure 1f**), where both fast and slow nucleotide exchange was observed. Even with high concentrations (3 mM) of magnesium fluoride, only about half of the amplitude corresponded to the slow phase, which indicates that magnesium fluoride alone was not sufficient to form a stable TSA complex under the conditions of our experiment. Further evidence was provided by two-dimensional NMR experiments in the presence of magnesium fluoride under aluminium-free conditions. Based on the similarity of $^{1}H_{\varepsilon}$-$^{15}N_{\varepsilon}$ correlation maps available for different states, these experiments indicated that nucleotides are bound but no transition state analog is formed under these conditions. However, in the presence of significantly less aluminium fluoride, the TSA complex was fully formed and it is characterized exclusively by slow nucleotide exchange kinetics and a long lifetime. Although the ADP:$AlF_x$:UMP (TSA) complex decays at a rate that is substantially lower than dissociation of individual nucleotides or even bi-substrate inhibitor, it only displays moderate affinity for $AlF_x$ ($K_D \approx 30$ μM; **Figure S2**). Initially this may seem surprising given it is considered to be a *bona fide* TSA, but one shall

not forget that this TSA "construct" consists of 3 individual molecules, which are not linked covalently, and that a substantial entropic penalty for assembly of the components has to be contoured by binding energy[6].

**4. Sample preparation for NMR experiments**

All NMR experiments were performed in buffer containing 50 mM Tris/HCl pH 7.5, 20 mM KCl, 2 mM DTE, 1 mM sodium azide. The $D_2O$ required for locking the NMR spectrometer was inserted into the NMR tube using an inset. This strategy has the advantage that splitting of peaks due to deuterium isotope shifts is avoided, thereby leading to higher quality NMR spectra.

| Nucleotide-free | $AP_5U$-bound | TSA-bound |
|---|---|---|
| 600 μM UmpK | 600 μM UmpK | 600 μM UmpK |
| | 750 μM $AP_5U$ | 1.5 mM ADP |
| | 3.0 mM $MgCl_2$ | 4.0 mM UMP |
| | | 1.5 mM $AlF_x$ |
| | | 3.0 mM $MgCl_2$ |

The $AlF_x$ stock solution was prepared from $AlCl_3$ and NaF in 1:5 molar ratio at pH 6.

**5. Chemical shift assignment of the arginine side-chains of UmpK:**

The general strategy was to make use of arginine-to-lysine mutations as suggested previously[7], in order to identify the eight arginine side-chains of UmpK one by one, simply by observing peaks disappearing in the NMR correlation spectra (**Figure S3**). Due to the nanomolar affinity of $AP_5U$, the "substrate-bound" $AP_5U$ state is resilient to arginine-to-lysine mutations and we successfully used the strategy of arginine-to-lysine mutation for the chemical shift assignment of both the $AP_5U$-bound state and the nucleotide-free state of UmpK. However, this approach fell short for the TSA state as most mutations impaired the formation of this sophisticated transition state complex. The arginine side-chain chemical shifts were therefore assigned via backbone resonances of the TSA state, which in turn were obtained using standard triple-resonance NMR experiments (HNCA[8], HN(CO)CA[8a, 8b], HNCO[8b, 8c], HN(CA)CO[8b, 9], HNCACB[10]). To aid the assignment procedure we also utilized specifically labelled samples [$^{13}C$,$^{15}N$-Arg; U-$^{12}C$,$^{14}N$]-UmpK and [$^{13}C$,$^{15}N$-Arg,Lys; U-$^{12}C$,$^{14}N$]-UmpK, in which only the arginine (and/or lysine) residues were labelled with $^{15}N$ and $^{13}C$. The arginine side-chain carbon chemical shifts were subsequently obtained by an HCCH-TOCSY[11] experiment and the link between backbone and side-chain assignment was

obtained by comparing the $^{13}C_\delta$ chemical shifts that are available from HNCA-type experiment via the magnetization transfers $^1H_\varepsilon \rightarrow {}^{15}N_\varepsilon \rightarrow {}^{13}C_\delta$.

Of note is that the side-chain of R127 forms a π-stacking interaction with the adenine moiety of the bound ligands, which may be the cause of the particularly low $^{13}C_\zeta$ chemical shift of this residue. In the nucleotide-free state, only R28 and R176, which are both integrated in hydrogen bonding networks, are visible in the proton-detected spectrum.

## 6. Nuclear spin-relaxation measurements:

***Overall correlation time $\tau_m$:*** For model-free analysis[12], the overall correlation time $\tau_m$ of the protein is required as an input, which we determined from $^{15}N$ backbone relaxation rates in the $AP_5U$ state. The obtained value of $\tau_m = (11.2 \pm 0.1)$ ns is in agreement with previous measurements of the highly homologous adenylate kinase in complex with $AP_5A$[13] and can also be assumed for the TSA complex since their overall structures are identical.

***Arginine side-chain spin-relaxation:*** Arginine side-chain $R_1(N_z)$ and $R_2(N_x)$ relaxation rates were obtained to gain access to the level of conformational heterogeneity of the catalytically essential arginine side-chains. Such measurements are possible from proton-detected spectra following standard procedures and also from the carbon-detected spectra employing techniques that were recently developed[7]. Specifically, the transverse relaxation rate $R_2(N_x)$ is obtained via the corresponding relaxation rate in the rotating frame. The direction of the effective field for the $^{15}N$ nucleus during the application of a spin-lock field, $\omega_{SL}$, along $\hat{x}$ is $\hat{z}' = \sin(\theta)\hat{x} + \cos(\theta)\hat{z}$, where $\hat{x}$ and $\hat{z}$ are direction vectors in the rotating frame, $\tan(\theta)=\omega_{SL}/\Omega_N$, and $\Omega_N$ is the offset of the $^{15}N$ nucleus from the RF carrier, respectively. Here $\omega_{SL} \approx 2\pi\times1500$ Hz to minimize the contributions from micro-millisecond exchange dynamics. The relaxation rate of the in-phase and anti-phase coherences in the rotating frame, which are used as input for the model-free analysis, are then given by[14],

$$R_{1\rho}(\mathrm{N_x}) = \cos^2(\theta)R_1(\mathrm{N_z}) + \sin^2(\theta)R_2(\mathrm{N_x}) \quad \text{(S1)}$$

$$R_{1\rho}(2\mathrm{C_z N_x}) = \cos^2(\theta)R_1(2\mathrm{C_z N_z}) + \sin^2(\theta)R_2(2\mathrm{C_z N_x}) \quad \text{(S2)}$$

The transverse relaxation rates, which form the basis for obtaining $S^2$ order parameters, can in some instances have small contributions from slower micro-millisecond exchange dynamics, even when obtained via relaxation rate in the rotating frame. We therefore also employed a set of exchange-free relaxation rates, $R_{dd}$, that yield the "uncontaminated" dipole-dipole $^{15}N$ transverse relaxation rate[15]. Because of the nature of carbon-detected experiments, the anti-

phase rates, $R_2(2C_zN_x)$ and $R_1(2C_zN_x)$ are obtained, however, as detailed previously the in-phase rates can easily be obtained by,

$$R_1(\mathrm{N_z}) \simeq R_1(2\mathrm{C_zN_z}) - R_1(\mathrm{C_z}) \qquad \text{(S3)}$$

and

$$R_2(\mathrm{N_x}) \simeq R_2(2\mathrm{C_zN_x}) - R_1(\mathrm{C_z}), \qquad \text{(S4)}$$

which leads to systematic errors less than 4.5% of the obtained rate. We assumed a uniform $R_1(C_z)$ of 0.5 $s^{-1}$ throughout the analysis based on data from $AP_5U$-bound UmpK.

For the nucleotide-free form of UmpK all active-site arginines give rise to strong peaks in the carbon-detected experiment, but are invisible in the proton-detected correlation maps. We therefore obtained the transverse relaxation rate of the arginine side-chains of nucleotide-free UmpK at a static magnetic field of 16.4 T using the newly developed carbon-detected experiments[7]. In contrast, in the TSA bound state most arginine peaks could be identified in the proton-detected spectra (with the exception of R148), while the carbon-detected spectra yielded no extra information. We therefore employed proton-detected relaxation experiments of $R_1$, $R_{1\rho}$ and $R_{dd}$ at 11.7 T for this state. In the $AP_5U$-bound state many arginine side-chains were visible in both, the proton- and carbon-detected spectra, but the two types of spectra also provide complementary information (e.g. R42 and R137 are only visible in carbon-detected spectra). Consequently, for the $AP_5U$ state both, proton- and carbon-detected relaxation experiments were performed at different magnetic field strengths. Specifically, carbon-detected relaxation experiments were performed at 11.7 T, 14.1 T, and 16.4 T, while proton-detected relaxation experiments were performed at 11.7 T. The obtained relaxation rates are listed in **Table S2**.

***Extracting motional parameters:*** As described previously[7, 12b, 15-16], for an $^1H_\varepsilon$-$^{15}N_\varepsilon$ spin-pair, the longitudinal relaxation rate, $R_1(N_z)$, the transverse relaxation rate, $R_2(N_x)$, and the dipole-dipole exchange-free transverse relaxation rate $R_{dd}$ can be expressed in terms of the spectral density function, $J(\omega)$:

$$R_1(\mathrm{N_z}) = \frac{1}{4}d^2\big(3J(\omega_\mathrm{N}) + J(\omega_\mathrm{H} - \omega_N) + 6J(\omega_\mathrm{H} + \omega_N)\big) + c^2J(\omega_\mathrm{N}) \qquad \text{(S5)}$$

$$R_2(\mathrm{N_x}) = \frac{1}{8}d^2(4J(0) + 3J(\omega_\mathrm{N}) + J(\omega_\mathrm{H} - \omega_N) + 6J(\omega_\mathrm{H}) + 6J(\omega_\mathrm{H} + \omega_N)) + \frac{1}{6}c^2(4J(0) + 3J(\omega_N)) + R_\mathrm{ex} \qquad \text{(S6)}$$

$$R_{dd} = \frac{1}{8}d^2\big(4J(0) - 3J(\omega_\mathrm{N}) + 0.5\,J(\omega_\mathrm{H} - \omega_N) - 3J(\omega_\mathrm{H}) + 3J(\omega_\mathrm{H} + \omega_N)\big) \qquad \text{(S7)}$$

where

$$c = \omega_N \, \Delta\sigma_N / \sqrt{3}$$
$$d = (\mu_0 h \gamma_N \, \gamma_H / 8\,\pi^2)\langle 1/r_{NH}^3 \rangle,$$

and $J(0)$, $J(\omega_N)$ and $J(\omega_H)$ are the values of the spectral density function evaluated at frequencies of 0 rad $s^{-1}$, $\omega_N$, and $\omega_H$, respectively, where $\omega_N$ is the $^{15}$N Larmor frequency and $\omega_H$ is the proton Larmor frequency. The contribution to the transverse relaxation that originates from fluctuations of the Zeeman Hamiltonian due to chemical exchange processes is $R_{ex}$. $\Delta\sigma$ is the chemical shift anisotropy (in ppm), assuming axial symmetry for the $^{15}$N chemical shift tensor ($\Delta\sigma_N = -114$ ppm for $^{15}N_\varepsilon$ of the arginine side-chain[17]), $\mu_0$ is the permeability of free space, $h$ is Planck's constant, $\gamma_H$ and $\gamma_N$ are the magnetogyric ratios of $^1$H and $^{15}$N, respectively, and $r_{NH}$ = 1.03 Å is the N-H bond length[16-17].
The spectral density function, $J(\omega)$, can be expressed in the model-free formalism[12a, 18] as a function of the generalized order parameter, $S^2$, the overall rotational correlation time of the protein, $\tau_m$, and a time-constant for the local motion, $\tau_e$.

$$J(\omega) = \frac{2}{5}\left(\frac{S^2\tau_m}{1+\omega^2\tau_m^2} + \frac{(1-S^2)\tau_{e\prime}}{1+\omega^2\tau_{e\prime}^2}\right) \qquad \text{(S8)}$$

where $\tau_{e\prime}^{-1} = \tau_m^{-1} + \tau_e^{-1}$ and $\tau_e$ is the correlation time for the local side-chain motion.

The exchange-free experiment which provides the $R_{dd}$ rate is feasible only in a proton-detected fashion, which excludes the nucleotide-free state from the more sophisticated analysis described in the following. In contrast, in the $AP_5U$-bound state many arginine side-chains are visible in both the proton-detected ($^{15}$N-$^1$H) and carbon-detected ($^{15}$N-$^{13}$C) spectra. Thus, we used this large dataset to check the robustness of the fitting procedure by including exclusively the proton- or carbon-detected data or forcing the exchange contribution $R_{ex}$ to be zero. We obtained consistent results in all cases. Furthermore, all local correlation times $\tau_e$ were below 1 ns, thus at least one order of magnitude faster than the overall tumbling described by $\tau_m$, which is a prerequisite for the applied model. It should also be mentioned that the contribution from chemical exchange ($R_{ex}$) is at most 20% of the respective $R_2$ value even at the highest magnetic field strength (**Table S3**).
Overall, proton- and carbon-detected relaxation experiments performed at the different magnetic field strengths for $AP_5U$-bound and TSA state were analyzed in a global fitting routine applying the model-free approach[12a, 18] to extract order parameter $S^2$, local correlation time $\tau_e$ and the exchange contribution $R_{ex}$ for each arginine side-chain.

**7. Estimation of conformational entropy from arginine side-chain order parameters:**

Side-chain order parameters have been shown to generally fall within bands[19] indicating the nature of motion that give raise to the order parameter, and side-chain order parameters between 0.3 and 0.7 indicate that one side-chain dihedral angle is involved in jumps between rotameric states[17, 19b, 19d], while order parameters above 0.7 indicate that there is no dihedral disorder. The order parameters obtained from $^{15}N_{\varepsilon}$ relaxation data of the active-site arginine residues are therefore directly related to the conformational heterogeneity of the catalytically essential chemical groups and therefore directly related to the conformational entropy of the active site.

We here base our estimations of conformational entropy from side-chain order parameters on the work by Wand and co-workers[19a, 19c], which was guided by work by Karplus[20], Akke *et al.*[21] and Yang & Kay[22]. In their models, the internal protein dynamics between disjoint states forms the basis for calculating the conformational entropy as:

$$S_{\mathrm{conf}} = \sum_i p_i S_i - k_{\mathrm{B}} \sum_i p_i \ln p_i \qquad \text{(S9)}$$

where $S_i$ is the intrinsic entropy in the $i$th state due to fast intra-well motions, $k_B$ is Boltzmann's constant, and $p_i$ is the population of the $i$th side-chain dihedral rotameric state. It was shown previously that the conformational entropy varies primarily through changes in the populations of rotameric states and that changes in the first term of Eq (S9) are small. This means, in turn, that the *change* in conformational entropy can be predicted from the *change* in population of distinct rotameric states, or to a good approximation from the number of side-chain dihedral angles significantly engaged in rotameric jumps.

To support the above approach is that it has been shown previously that the conformational entropy associated with the general restriction of one rotor (dihedral angle) is 2–5 kJ/mol[23]. In the limit, where one rotameric state is populated to 90% and one other state populated 10% gives a conformational entropy change of 0.8 kJ/mol relative to a rigid conformation[19d]. With focus on the arginine side-chain, the $\chi_3$ angle populates in the random coil gauche+ with 22%, trans with 50%, and gauche− with 28%[24], which leads to a change of conformational entropy of $-T\Delta S = -2.6$ kJ/mol compared to a rigid $\chi_3$ angle. Moreover, for an arginine side-chain with random coil populations[24] one can calculate according to the second term of Eq (S9) that $-T\Delta S = -8.1$ kJ/mol.

The basis for our simulation is the 34 states of the arginine side-chain that have been assigned previously[24]; $\{s_i\}_{i\leq 34}$. In a Monte Carlo approach we first assigned the number of

states, $n_s$, being sampled and subsequently assigned, randomly, the populations of the $n_s$ sampled states. In order to calculate the $^1H_\varepsilon$-$^{15}N_\varepsilon$ order parameters for different dynamic distributions, the 34 arginine side-chain structures were constructed and the angles between the $^1H_\varepsilon$-$^{15}N_\varepsilon$ bond vectors in the structures were calculated. The order parameter can subsequently be calculated from the equilibrium populations[18] according to

$$S^2 = \int\int \mathrm{d}\Omega_1 \mathrm{d}\Omega_2\, p_{eq}(\Omega_1) p_{eq}(\Omega_2) P_2(\cos\theta_{12}) \qquad \text{(S10)}$$

where $P_2(z)$ is the second Legendre polynomial, $\Omega_i$ describes the orientation of the inter-nuclear N-H vector $\mathbf{v}_i$, and $\theta_{12}$ is the angle between the vectors $\mathbf{v}_1$ and $\mathbf{v}_2$, that is, $\mathbf{v}_1 \bullet \mathbf{v}_2 = \cos(\theta_{12})$. For the discrete scenario considered here, where $n_s$ of the 34 discrete states are sampled, the order parameter is calculated as

$$S^2_{dih} = \sum_{i,j\,\in\{s_i\}} p_i p_j P_2(\cos\theta_{ij}) \qquad \text{(S11)}$$

For each combination of the populations, the conformational entropy was calculated according to Term 2 of Eq S9 and the order parameter calculated according to Eq S11. The result of a Monte Carlo simulation involving $8\times10^6$ randomly selected $n_s$ and populations is shown in **Figure 4**. Also shown is the correlation between conformational entropy and $^1H_\varepsilon$-$^{15}N_\varepsilon$ order parameters obtained previously for RNaseH using molecular dynamics[17]. Since we are here interested in changes in conformational entropy and not the absolute entropy, an offset was added to the entropies obtained previously by Trbovic *et al.* Such an offset stems (*i*) from the method of integration used previously and (*ii*) from the fact that the estimations by Trbovic *et. al.* includes all degrees of freedoms, whereas the estimations made here are calculated such that a rigid and single dihedral-angle conformation ($S^2_{\text{dih}}$=1.0) corresponds to an entropy of 0 kJ/mol. The offset, *c*, between the data by Trbovic *et al.* and our calculations was determined by minimizing the target function

$$\chi^2(c) = \sum_{residue}\sum_{i}\{S_{calc,i}(|\,S^2_{calc,i} - S^2_{Trbovic}\,| < 0.03) - S_{Trbovic} + c\}^2\,,$$

where the sum over residues are the $\{S^2_{Trbovic}, S_{Trbovic}\}$ points and the sum over $i$ is over the simulated points for which $|S^2_{Trbovic} - S^2_{calc,i}| < 0.03$. Excluding the outlier at $S^2_{Trbovic} \sim 0.9$ (Figure 4) gives an offset of 40.3 kJ/mol, whereas including all $\{S^2_{Trbovic}, S_{Trbovic}\}$ points gives an offset of 39.8 kJ/mol. The offset of 39.8 kJ/mol was used for the correlation shown in Figure 4. After applying the offset, there is generally a good agreement between the trend

obtained previously from molecular dynamics simulations and the distribution obtained here from Monte Carlo simulations.

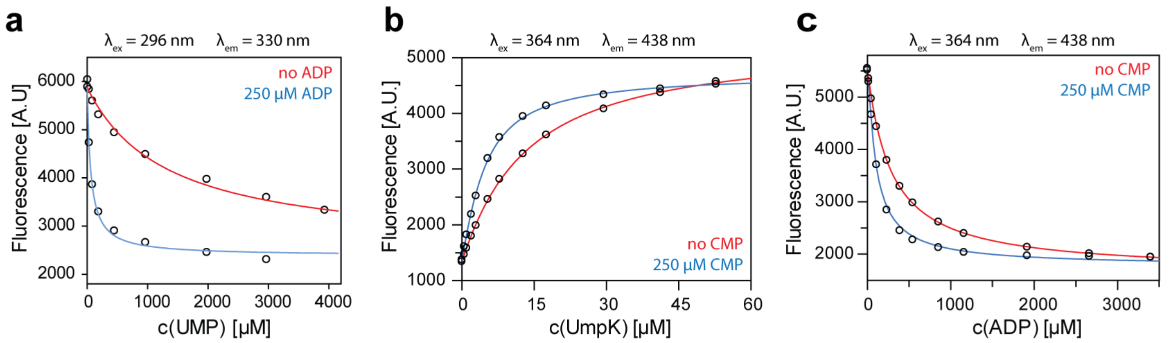


**Figure S1. Cooperative nucleotide binding. (a)** UMP binding to UmpK wild-type in the presence (blue) and absence (red) of 250 μM ADP monitored by tryptophan fluorescence. The titration was performed at c(UmpK) = 5 μM. **(b)** MANT-ADP binding to UmpK wild-type in the presence (blue) and absence (red) of 250 μM CMP monitored by MANT fluorescence. The titration was performed at c(MANT-ADP) = 3 μM. **(c)** ADP binding to UmpK wild-type in the presence (blue) and absence (red) of 250 μM CMP monitored by MANT fluorescence. The displacement titration was performed at c(UmpK) = 25 μM and c(MANT-ADP) = 5 μM. $K_D$(ADP) was determined by fitting with the cubic equation for binding of competing ligands[3]. All binding parameters obtained from these experiments are listed in **Table S1.** (A.U. = arbitrary units)

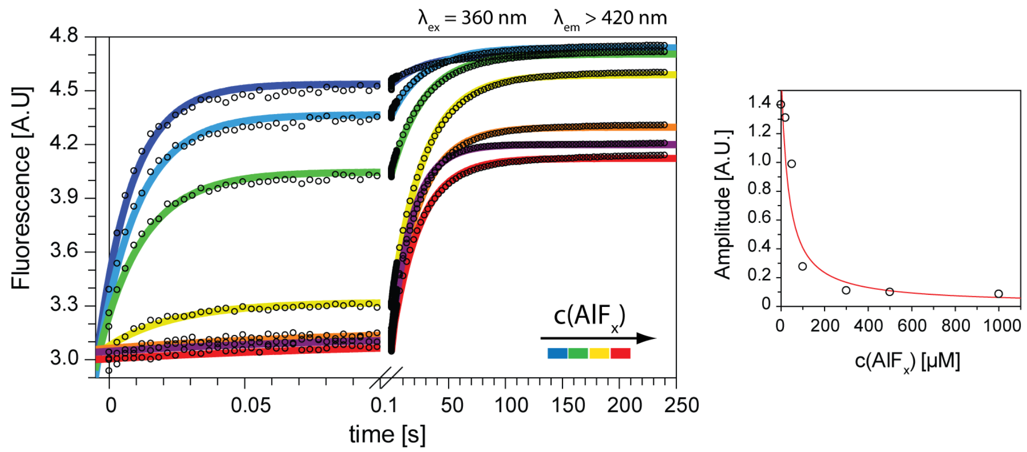


**Figure S2. Aluminium fluoride ($AlF_x$) titration**. Nucleotide exchange kinetics were measured by stopped flow to characterize the TSA complex. UmpK was incubated with UMP and ADP in the presence of increasing concentrations of aluminium fluoride (0 µM – dark blue, 20 µM – light blue, 50 µM – green, 100 µM – yellow, 300 µM orange, 500 µM – red, 1000 µM – purple) and then mixed rapidly with the competing ligand MANT-$AP_5$A-MANT. 3 mM $MgCl_2$ was present in all buffers. A bi-exponential decay was fitted and the amplitude of the fast phase was used to estimate an apparent binding affinity for $AlF_x$ ($K_D \approx 30$ µM). (A.U. = arbitrary units)

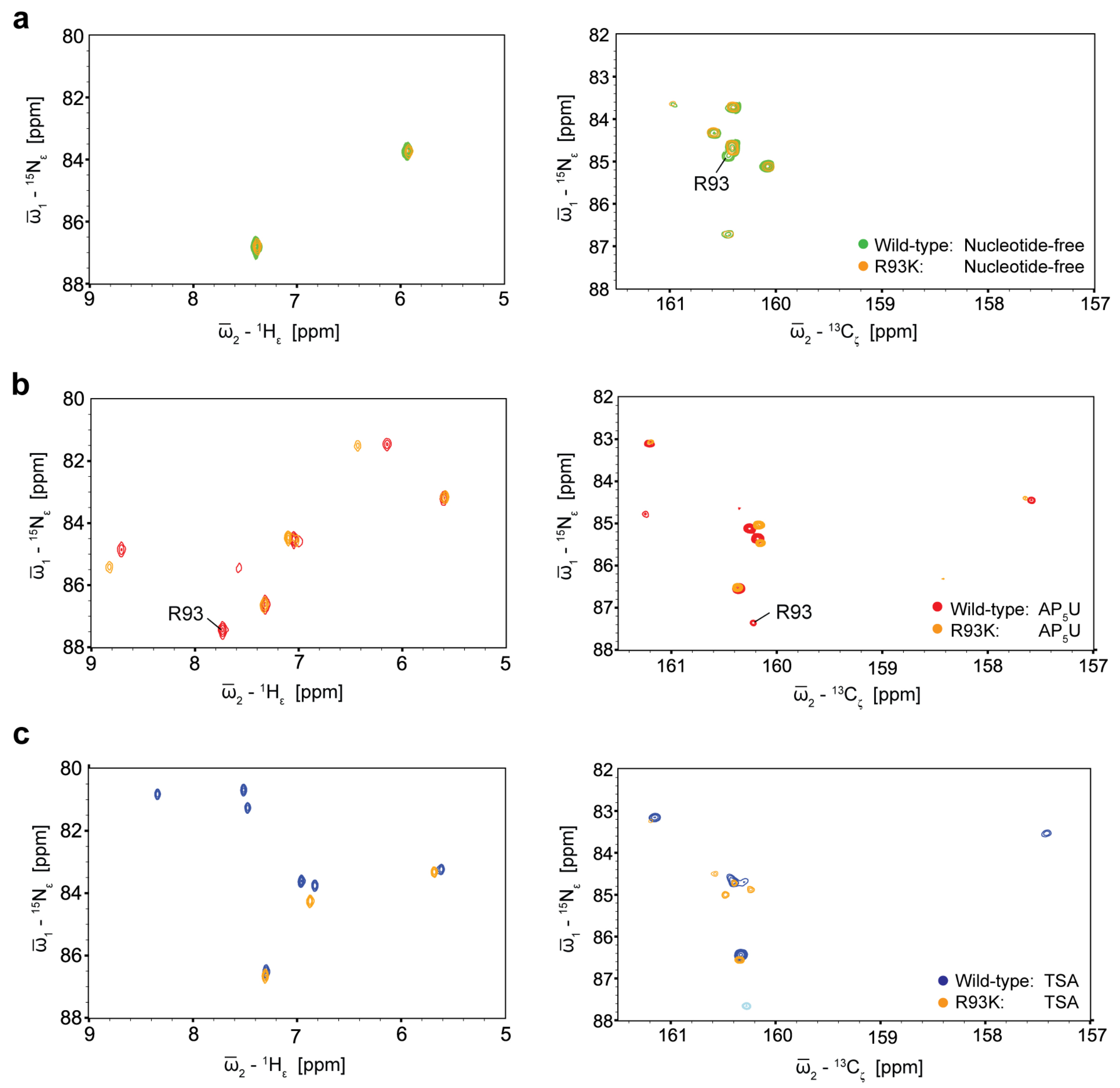


**Figure S3. Representative NMR correlation spectra of arginine-to-lysine mutants of UmpK.** An overlay of arginine side-chain spectra from UmpK R93K and wild-type is shown **(a)** in the nucleotide-free state, **(b)** in the AP$_5$U state, and **(c)** in the 'TSA complex'. In **(a)** and **(b)**, one resonance clearly disappears due to the mutation, while all other resonances are not affected significantly. This allowed the assignment of the arginine side-chain resonances in both states. For R42 and R137 in the AP$_5$U state, a slight ambiguity remained due to both, the close spatial proximity of these arginine side chains in the structure as well as the proximity of these peaks in the spectra. Mutation of one residue (e.g. R42K) led to substantial peak movement of the neighboring side chain (e.g. R137). In this case, we chose to assign the respective residue based on the shortest distance of the cross-peak in question to the respective cross-peak in the carbon-detected spectrum of wild-type UmpK in the AP$_5$U state. In **(c)** the spectra of the arginine-to-lysine mutant were widely different from UmpK wild-type, thereby indicating that the TSA complex is not formed in the presence of the mutation. Here, the assignment was obtained via the backbone resonances as described in the **Online Methods**.

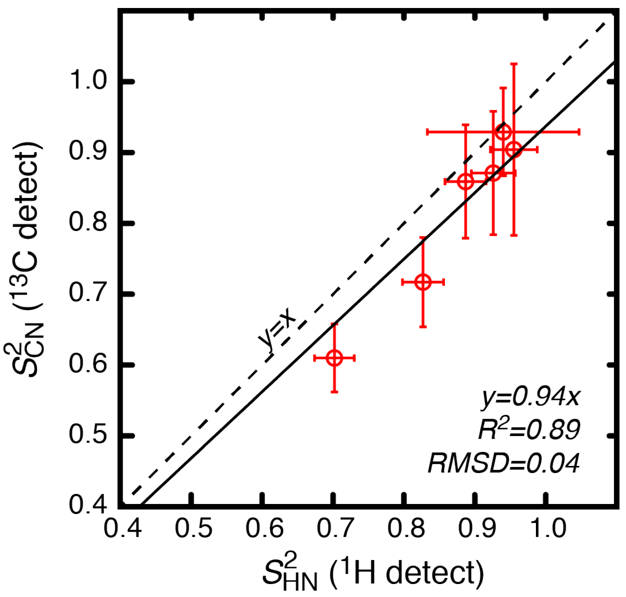


**Figure S4. Consistency of derived order parameters.** For six of the arginine side chains of the substrate-like sample, UmpK:AP$_5$U, the relaxation rates can be obtained from both the $^1$H-detected and the $^{13}$C-detected experiments. Thus, order parameters, $S^2$, can be determined separately from the two types of experiments and compared in order to provide assurance of the consistency of the obtained parameters. Above the $S^2$ derived from the carbon-detected experiments, $S^2_{CN}$, are compared with those derived from proton-detected $^1$H–$^{15}$N experiments, $S^2_{HN}$ . For both $S^2_{CN}$ and $S^2_{HN}$ the parameters were determined using all available rates within each type of experiment and it was for this comparison assumed that the contribution from chemical exchange processes is negligible. *RMSD* = root-mean-square-deviation, and $R^2$ is the Pearson coefficient of linear correlation. The correlation obtained here for the UmpK:AP$_5$U is very similar to that obtained previously.[7]

**Table S1. Cooperative nucleotide binding.** Binding affinities of UMP/CMP kinase (UmpK) determined by fluorescence titrations.

| (in µM) | **UmpK wild-type (5 mM $MgCl_2$)** | | | **UmpK wild-type (Mg-free)** | | | **UmpK R93K (5 mM $MgCl_2$)** | |
|---|---|---|---|---|---|---|---|---|
| | ***Alone*** | ***in the presence of 250 µM*** | | ***Alone*** | ***in the presence of 250 µM*** | | ***Alone*** | ***in the presence of 250 µM ADP*** |
| **$K_D$(UMP)** | 1500 ± 300 | 62 ± 7 | ADP | 2000 ± 450 | 1400 ± 250 | ADP | 3500 ± 1200 | 800 ± 100 |
| **$K_D$(UDP)** | 600 ± 70 | 60 ± 14 | ADP | 110 ± 13 | 400 ± 45 | ADP | - | - |
| **$K_D$(CMP)** | 77 ± 11 | 23 ± 3 | ADP | 150 ± 17 | 205 ± 20 | ADP | - | - |
| **$K_D$(CDP)** | 66 ± 11 | 17 ± 3 | ADP | 13 ± 2 | 64 ± 6 | ADP | - | - |
| **$K_D$(MANT-ADP)** | 11.7 ± 0.3 | 3.2 ± 0.2 | CMP | 1.9 ± 0.1 | 2.6 ± 0.1 | CMP | 7.6 ± 0.2 | - |
| **$K_D$(ADP)** | 80 ± 2 | 10 ± 1 | CMP | 11 ± 0.3 | 49 ± 0.5 | CDP | 31 ± 2 | - |
| **$K_D$(ATP)** | 51 ± 2 | 7.3 ± 0.4 | CMP | 13 ± 1 | 68 ± 3 | CMP | - | - |
| **$K_D$($AP_5U$)** | 0.0002 ± 0.0010 | - | | 0.0015 ± 0.0035 | - | | 0.0015 ± 0.0037 | - |

**Table S2. $^{15}N_{\varepsilon}$ relaxation data of UmpK from $^{1}H$-detected and $^{13}C$-detected experiments at different magnetic field strengths.**
The values served as the input for the global data fitting using the model-free approach. (SE = standard error)

| (in $s^{-1}$) | **$^{1}H_{\varepsilon}$-$^{15}N_{\varepsilon}$ (500 MHz)** | | | | | | **$^{13}C_{\zeta}$-$^{15}N_{\varepsilon}$ (500 MHz)** | | | | | | **$^{13}C_{\zeta}$-$^{15}N_{\varepsilon}$ (600 MHz)** | | | | | | **$^{13}C_{\zeta}$-$^{15}N_{\varepsilon}$ (700 MHz)** | | | | | |
|---|---|---|---|---|---|---|---|---|---|---|---|---|---|---|---|---|---|---|---|---|---|---|---|---|
| **Nucleotide-free** | | | | | | | | | | | | | | | | | | | | | | | | |
| **# Arginine** | | | | | | | **$^{15}N_{\varepsilon}$-$R_1$** | SE | **$^{15}N_{\varepsilon}$-$R_2$** | SE | **$R_1(C_Z)$** | SE | | | | | | | **$^{15}N_{\varepsilon}$-$R_1$** | SE | **$^{15}N_{\varepsilon}$-$R_2$** | SE | **$R_1(C_Z)$** | SE |
| **R28** | | | | | | | - | - | 9.64 | 1.64 | 0.29 | 0.33 | | | | | | | 1.27 | 0.42 | 11.83 | 0.95 | 0.10 | 0.37 |
| **R42** | | | | | | | - | - | 3.60 | 0.85 | 0.67 | 0.08 | | | | | | | 1.36 | 0.18 | 2.97 | 0.32 | 0.50 | 0.20 |
| **R93** | | | | | | | - | - | 4.70 | 0.74 | 1.12 | 0.63 | | | | | | | 1.86 | 0.58 | 4.49 | 1.21 | 0.68 | 0.06 |
| **R127** | | | | | | | - | - | 4.12 | 1.63 | 0.46 | 0.10 | | | | | | | 1.14 | 0.40 | 6.73 | 0.79 | 0.58 | 0.35 |
| **R131** | | | | | | | - | - | 3.76 | 1.03 | 0.65 | 0.21 | | | | | | | 1.15 | 0.20 | 3.80 | 0.56 | 0.45 | 0.12 |
| **R137** | | | | | | | - | - | 1.46 | 0.36 | 0.14 | 0.26 | | | | | | | 0.78 | 0.06 | 2.27 | 0.56 | 0.42 | 0.16 |
| **R148** | | | | | | | - | - | 6.22 | 1.29 | 0.60 | 0.03 | | | | | | | 1.37 | 0.14 | 5.52 | 0.79 | 0.49 | 0.13 |
| **R176** | | | | | | | - | - | 9.64 | 1.64 | 0.04 | 0.32 | | | | | | | 0.37 | 0.51 | 17.25 | 7.04 | 1.81 | 0.39 |
| **$AP_5U$** | | | | | | | | | | | | | | | | | | | | | | | | |
| **# Arginine** | **$^{15}N_{\varepsilon}$-$R_1$** | SE | **$^{15}N_{\varepsilon}$-$R_2$** | SE | **$^{15}N_{\varepsilon}$-$R_{dd}$** | SE | **$^{15}N_{\varepsilon}$-$R_1$** | SE | **$^{15}N_{\varepsilon}$-$R_2$** | SE | **$R_1(C_Z)$** | SE | **$^{15}N_{\varepsilon}$-$R_1$** | SE | **$^{15}N_{\varepsilon}$-$R_2$** | SE | **$R_1(C_Z)$** | SE | **$^{15}N_{\varepsilon}$-$R_1$** | SE | **$^{15}N_{\varepsilon}$-$R_2$** | SE | **$R_1(C_Z)$** | SE |
| **R28** | 1.05 | 0.06 | 9.15 | 0.18 | 10.16 | 2.66 | 1.69 | 0.14 | 9.16 | 0.63 | 0.44 | 0.04 | 0.86 | 0.14 | 8.57 | 1.11 | 0.57 | 0.07 | - | - | 7.94 | 0.56 | - | - |
| **R42** | - | - | - | - | - | - | 1.87 | 0.25 | 9.08 | 0.84 | 0.65 | 0.08 | 1.23 | 0.12 | 7.89 | 0.73 | 0.76 | 0.11 | - | - | 6.36 | 0.35 | - | - |
| **R93** | 1.20 | 0.04 | 13.01 | 0.19 | 9.48 | 1.30 | 1.67 | 0.12 | 12.73 | 4.38 | 0.29 | 0.25 | 0.38 | 0.58 | 12.09 | 2.39 | 0.43 | 0.19 | - | - | 11.5 | 1.43 | - | - |
| **R127** | 1.19 | 0.01 | 11.60 | 0.32 | 10.12 | 0.76 | 1.50 | 0.31 | 12.96 | 1.63 | 0.18 | 0.15 | 1.38 | 0.22 | 11.84 | 1.34 | 0.23 | 0.12 | - | - | 13.11 | 0.95 | - | - |
| **R131** | 1.25 | 0.10 | 14.72 | 2.24 | 10.22 | 6.40 | 2.64 | 0.51 | 16.01 | 0.90 | 0.64 | 0.13 | 1.33 | 0.35 | 9.14 | 2.86 | 0.57 | 0.26 | - | - | - | - | - | - |
| **R137** | - | - | - | - | - | - | 2.00 | 0.29 | 8.45 | 0.43 | 0.79 | 0.30 | 1.08 | 0.19 | 9.51 | 1.19 | 0.44 | 0.09 | - | - | 8.02 | 0.61 | - | - |
| **R148** | 1.25 | 0.04 | 12.97 | 0.41 | 14.65 | 2.05 | 2.73 | 1.49 | 18.17 | 2.78 | 0.29 | 0.42 | - | - | - | - | - | - | - | - | - | - | - | - |
| **R176** | 1.17 | 0.03 | 10.67 | 0.30 | 10.31 | 1.64 | 1.84 | 0.38 | 8.76 | 0.63 | 0.47 | 0.15 | 0.68 | 0.18 | 13.69 | 0.84 | 0.37 | 0.11 | - | - | 8.59 | 2.38 | - | - |
| **TSA** | | | | | | | | | | | | | | | | | | | | | | | | |
| **# Arginine** | **$^{15}N_{\varepsilon}$-$R_1$** | SE | **$^{15}N_{\varepsilon}$-$R_2$** | SE | **$^{15}N_{\varepsilon}$-$R_{dd}$** | SE | | | | | | | | | | | | | | | | | | |
| **R28** | 1.08 | 0.04 | 8.28 | 0.32 | - | - | | | | | | | | | | | | | | | | | | |
| **R42** | 1.16 | 0.03 | 11.02 | 0.36 | 9.44 | 0.64 | | | | | | | | | | | | | | | | | | |
| **R93** | 1.19 | 0.04 | 12.74 | 0.58 | 8.49 | 1.15 | | | | | | | | | | | | | | | | | | |
| **R127** | 1.16 | 0.01 | 11.95 | 0.23 | 8.53 | 0.52 | | | | | | | | | | | | | | | | | | |
| **R131** | 1.09 | 0.05 | 12.51 | 0.39 | 8.25 | 0.89 | | | | | | | | | | | | | | | | | | |
| **R137** | 1.24 | 0.06 | 12.10 | 0.45 | 10.15 | 1.73 | | | | | | | | | | | | | | | | | | |
| **R148** | - | - | - | - | - | - | | | | | | | | | | | | | | | | | | |
| **R176** | 1.12 | 0.02 | 11.11 | 0.32 | 8.03 | 0.62 | | | | | | | | | | | | | | | | | | |

**Table S3. Order parameters $S^2$, local correlation times $\tau_e$ and exchange contributions $R_{ex}$ (at 500 MHz) obtained from global fitting of $^{15}N_\varepsilon$ relaxation data using the model-free approach for UmpK arginine side chains in the nucleotide-free state, AP$_5$U complex and TSA complex.**
(SE = standard error)

| # Arginine | $S^2$ | SE | $\tau_e$ (ns) | SE | $R_{ex}$ ($s^{-1}$) | SE |
|---|---|---|---|---|---|---|
| **Nucleotide-free** | | | | | | |
| **R28** | 0.74 | 0.09 | 2.24 | 1.16 | - | - |
| **R42** | 0.11 | 0.05 | 0.71 | 0.32 | - | - |
| **R93** | 0.12 | 0.09 | 1.39 | 0.74 | - | - |
| **R127** | 0.37 | 0.11 | 0.63 | 0.99 | - | - |
| **R131** | 0.18 | 0.05 | 0.52 | 0.30 | - | - |
| **R137** | 0.08 | 0.05 | 0.19 | 0.15 | - | - |
| **R148** | 0.31 | 0.08 | 0.89 | 0.66 | - | - |
| **R176** | - | - | - | - | - | - |
| **AP$_5$U** | | | | | | |
| **R28** | 0.67 | 0.06 | 0.08 | 0.05 | ~0 | - |
| **R42** | 0.47 | 0.11 | 0.21 | 0.27 | - | - |
| **R93** | 0.88 | 0.07 | <0.02 | 0.11 | 0.87 | 0.15 |
| **R127** | 0.89 | 0.04 | <0.02 | 0.08 | 0.13 | 0.16 |
| **R131** | 0.96 | 0.20 | <0.02 | 1.14 | ~0 | - |
| **R137** | 0.59 | 0.09 | 0.06 | 0.21 | - | - |
| **R148** | 0.93 | 0.11 | <0.02 | 0.19 | 0.79 | 0.31 |
| **R176** | 0.79 | 0.08 | 0.07 | 0.07 | ~0 | - |
| **TSA** | | | | | | |
| **R28** | 0.62 | 0.03 | 0.13 | 0.04 | - | - |
| **R42** | 0.85 | 0.04 | <0.02 | 0.06 | ~0 | - |
| **R93** | 0.82 | 0.08 | 0.07 | 0.10 | 2.06 | 0.15 |
| **R127** | 0.82 | 0.04 | 0.03 | 0.05 | 1.25 | 0.12 |
| **R131** | 0.79 | 0.05 | <0.02 | 0.06 | 2.25 | 0.07 |
| **R137** | 0.90 | 0.10 | <0.02 | 0.15 | 0.35 | 0.35 |
| **R148** | - | - | - | - | - | - |
| **R176** | 0.77 | 0.05 | 0.05 | 0.05 | 1.01 | 0.16 |

**References**


[1] T. Lorenz, J. Reinstein, *J Mol Biol* **2008**, *381*, 443-455.
[2] J. Reinstein, I. R. Vetter, I. Schlichting, P. Rosch, A. Wittinghofer, R. S. Goody, *Biochemistry* **1990**, *29*, 7440-7450.
[3] S. H. Thrall, J. Reinstein, B. M. Wohrl, R. S. Goody, *Biochemistry* **1996**, *35*, 4609-4618.
[4] I. Schlichting, J. Reinstein, *Nat Struct Biol* **1999**, *6*, 721-723.
[5] N. J. Baxter, G. M. Blackburn, J. P. Marston, A. M. Hounslow, M. J. Cliff, W. Bermel, N. H. Williams, F. Hollfelder, D. E. Wemmer, J. P. Waltho, *J Am Chem Soc* **2008**, *130*, 3952-3958.
[6] M. I. Page, W. P. Jencks, *Proc Natl Acad Sci U S A* **1971**, *68*, 1678-1683.
[7] N. D. Werbeck, J. Kirkpatrick, D. F. Hansen, *Angew Chem Int Ed Engl* **2013**, *52*, 3145-3147.
[8] a) S. Grzesiek, A. Bax, *J Magn Reson* **1992**, *96*, 432-440; b) L. E. Kay, G. Y. Xu, T. Yamazaki, *J Magn Reson* **1994**, *A109*, 129-133; c) J. Schleucher, M. Sattler, C. Griesinger, *Angew Chem Int Ed Engl* **1993**, *32*, 1489-1491.
[9] R. T. Clubb, V. Thanabal, G. Wagner, *J Magn Reson* **1992**, *97*, 213-217.
[10] a) D. R. Muhandiram, L. E. Kay, *J Magn Reson* **1994**, *B103*, 203-216; b) M. Wittekind, L. Mueller, *J Magn Reson* **1993**, *B101*, 201-205.
[11] L. E. Kay, G. Y. Xu, A. U. Singer, D. R. Muhandiram, J. D. Formankay, *J Magn Reson* **1993**, *B101*, 333-337.
[12] a) G. Lipari, A. Szabo, *J Am Chem Soc* **1982**, *104*, 4546-4559; b) L. E. Kay, D. A. Torchia, A. Bax, *Biochemistry* **1989**, *28*, 8972-8979.
[13] Y. E. Shapiro, M. A. Sinev, E. V. Sineva, V. Tugarinov, E. Meirovitch, *Biochemistry* **2000**, *39*, 6634-6644.
[14] D. M. Korzhnev, M. Billeter, A. S. Arseniev, V. Y. Orekhov, *Progress in Nuclear Magnetic Resonance Spectroscopy* **2001**, *38*, 197-266.
[15] D. F. Hansen, D. Yang, H. Feng, Z. Zhou, S. Wiesner, Y. Bai, L. E. Kay, *J Am Chem Soc* **2007**, *129*, 11468-11479.
[16] A. G. Palmer, 3rd, *Annu Rev Biophys Biomol Struct* **2001**, *30*, 129-155.
[17] N. Trbovic, J. H. Cho, R. Abel, R. A. Friesner, M. Rance, A. G. Palmer, 3rd, *J Am Chem Soc* **2009**, *131*, 615-622.
[18] G. Lipari, A. Szabo, *J Am Chem Soc* **1982**, *104*, 4559-4570.
[19] a) K. K. Frederick, M. S. Marlow, K. G. Valentine, A. J. Wand, *Nature* **2007**, *448*, 325-329; b) K. A. Sharp, V. Kasinath, A. J. Wand, *Proteins* **2014**, *82*, 2106-2117; c) A. J. Wand, V. R. Moorman, K. W. Harpole, *Top Curr Chem* **2013**, *337*, 69-94; d) R. B. Best, J. Clarke, M. Karplus, *J Am Chem Soc* **2004**, *126*, 7734-7735.
[20] M. Karplus, T. Ichiye, B. M. Pettitt, *Biophys J* **1987**, *52*, 1083-1085.
[21] M. Akke, R. Brueschweiler, A. G. Palmer, *J Am Chem Soc* **1993**, *115*, 9832-9833.
[22] D. Yang, L. E. Kay, *J Mol Biol* **1996**, *263*, 369-382.
[23] a) P. Schmid, D. Griller, K. U. Ingold, *International Journal of Chemical Kinetics* **1979**, *11*, 333-338; b) M. S. Searle, D. H. Williams, *Nucleic Acids Res* **1993**, *21*, 2051-2056.
[24] S. C. Lovell, J. M. Word, J. S. Richardson, D. C. Richardson, *Proteins* **2000**, *40*, 389-408.
